# Rare primary anorectal malignant melanoma presenting with painless hematochezia: a case report

**DOI:** 10.3389/fonc.2025.1640063

**Published:** 2025-08-20

**Authors:** Yuchun Zhong, Yueji Zou, Long Peng, Xiaoyun Hu, Wei Xu

**Affiliations:** ^1^ Department of General Surgery, The Second Affiliated Hospital of Nanchang University, Nanchang, Jiangxi, China; ^2^ The Institute of Translational Medicine, The Second Affiliated Hospital of Nanchang University, Nanchang University, Nanchang, Jiangxi, China; ^3^ Health Examination Center, The Second Affiliated Hospital of Nanchang University, Nanchang University, Nanchang, Jiangxi, China

**Keywords:** case report, primary anorectal malignant melanoma, hematochezia, laparoscopic-assisted abdominoperineal resection, treatment strategies

## Abstract

**Background:**

Anorectal malignant melanoma (ARMM) is an exceedingly rare and highly aggressive malignancy characterized by low prevalence, high misdiagnosis rates, and frequent recurrence/metastasis.

**Case report:**

This report details the case of a 51-year-old woman presenting with persistent bright red blood in her stool. Digital rectal examination revealed a firm, spherical mass approximately 4 cm from the anal verge. Colonoscopy identified a pedunculated polypoid lesion (~2.5 cm in diameter) near the anorectal junction. Based on clinical symptoms, physical findings, and endoscopic features, a high suspicion of rectal cancer was initially raised. However, subsequent histopathological evaluation of biopsy specimens revealed immunohistochemical positivity for MelanA, S100, and Ki-67 (~30%), suggesting a probable diagnosis of malignant melanoma. After completing preoperative contrast-enhanced abdominal CT and pelvic MRI examinations and excluding surgical contraindications, the patient underwent laparoscopic-assisted abdominoperineal resection (Miles procedure) and postoperative adjuvant therapy with toripalimab. Moreover, no signs of recurrence were found during follow-up over 3 months postoperatively.

**Conclusion:**

This case underscores that ARMM can be clinically indistinguishable from rectal carcinoma, posing a high risk of misdiagnosis. It highlights the critical role of histopathology and immunohistochemistry (IHC) in definitive differentiation, emphasizing the necessity of accurate diagnosis through IHC. Finally, it demonstrates the evolving treatment paradigm from extensive surgery toward a multidisciplinary approach integrating radical resection with adjuvant immunotherapy, reflecting advances in molecular insights and ameliorated outcomes.

## 1 Introduction

Primary anorectal malignant melanoma (ARMM) is an exceptionally rare and aggressive malignancy, accounting for <0.1% of all anorectal cancers (1). It represents one of the three most common sites for mucosal melanomas, following the head/neck and urogenital regions (2, 3).

ARMM mimics benign conditions such as hemorrhoids or polyps, leading to misdiagnosis in 30%–50% of cases (4). Furthermore, due to its aggressive nature, approximately 30% of patients present with distant metastases at initial diagnosis (5). Consequently, low prevalence, frequent misdiagnosis, and rapid progression result in advanced-stage disease at diagnosis, with a dismal 5-year survival rate of <20% (5, 6). Specifically, once ARMM is misdiagnosed or diagnosed too late, the probability of liver, lung, and bone metastases for the patient significantly increases, leading to a continuous deterioration of the optimal treatment plan and severely reducing the long-term prognosis of the patient (7). In this context, we present a case that illustrates the diagnostic challenges of early-stage ARMM mimicking rectal carcinoma and discuss the surgical and adjuvant therapeutic approaches.

## 2 Case presentation

**Figure 1:**
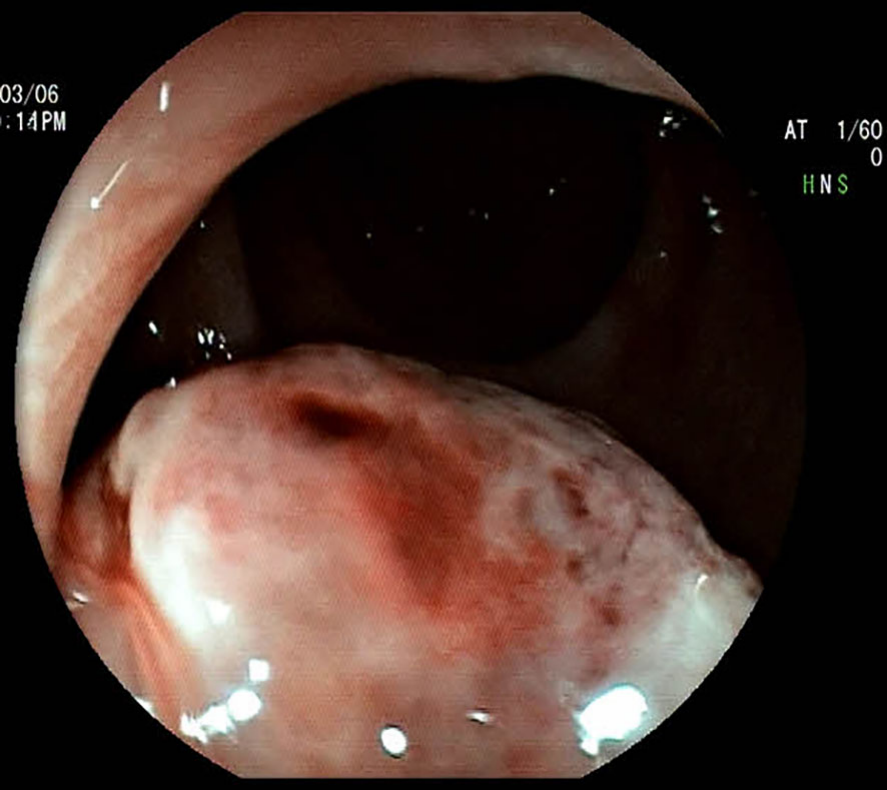
Colonoscopic image of the mass, demonstrating a spherical, elevated lesion with an eroded surface located approximately 3.6 cm from the anal verge.

A 51-year-old woman presented to our hospital with a 1-month history of bright red blood in her stool. Hematochezia was recurrent, with blood mixed with the feces. Associated symptoms included increased bowel frequency (from 1 to 6–7 times daily) and mild abdominal distension relieved by defecation or flatulence. No fever, dizziness, palpitations, nausea, vomiting, or abdominal pain was reported. Digital rectal examination in the knee-chest position identified a firm, spherical mass ~4 cm from the anal verge, with a rough, ulcerated surface occupying half the rectal circumference; the examining glove was stained with blood upon withdrawal. Colonoscopy revealed a pedunculated polypoid lesion (~2.5 cm in diameter) near the anorectal junction, with surface erosion (**Figure 1**). Based on the patient’s symptoms, physical examination, and colonoscopy findings, an initial diagnosis of rectal cancer was considered. The patient denied prior gastrointestinal inflammatory or neoplastic diseases, significant personal or family medical history, or relevant travel exposures.

### 2.1 Relevant medical laboratory examination

On admission, the patient’s vital signs were stable: body temperature was 36.7°C, blood pressure was 117/86 mmHg, and pulse rate was 75 beats per minute. Physical examination revealed no significant abnormalities in the head, neck, skin, mucous membranes, or superficial lymph nodes. The abdomen was soft without tenderness, rebound tenderness, muscle guarding, or palpable masses.

### 2.2 Laboratory investigations

Blood tests revealed the following: white blood cell count 5.69 × 10^9^/L, red blood cell count 3.4 × 10^12^/L, and hemoglobin 97 g/L. Tumor markers included alpha-fetoprotein (AFP: 3.6 ng/mL), carcinoembryonic antigen (CEA: 0.63 ng/mL), and carbohydrate antigen 19-9 (CA19-9: 7.09 U/mL), all within normal reference ranges.

### 2.3 Histopathological and immunohistochemical analysis

**Figure 2:**
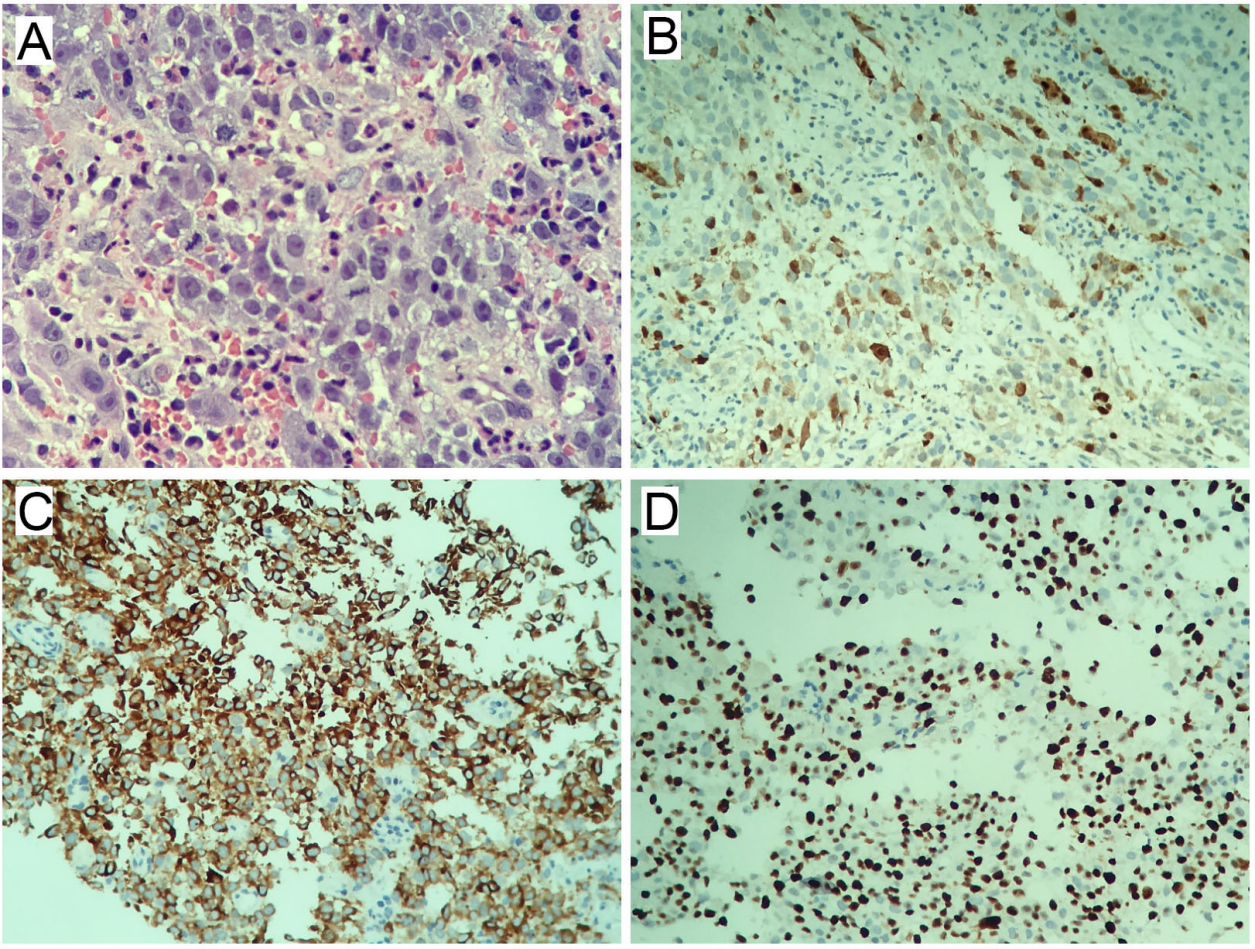
Histopathological and immunohistochemical staining. **(A)** Hematoxylin and eosin (H&E) staining (×400). **(B)** MelanA immunohistochemistry (IHC) (×200). **(C)** S100 immunohistochemistry (IHC) (×200). **(D)** Ki-67 immunohistochemistry (IHC) (×200).

Biopsy histopathology confirmed malignant melanoma (**Figure 2**). HE staining revealed disorganized tissue architecture, with neoplastic cells exhibiting enlarged, irregular nuclei, frequent mitotic figures, and uneven distribution (**Figure 2A**). Immunohistochemistry demonstrated MelanA positivity (**Figure 2B**), S100 positivity (**Figure 2C**), and Ki-67 expression in approximately 30% of tumor cells (**Figure 2D**).

### 2.4 Imaging findings

**Figure 3:**
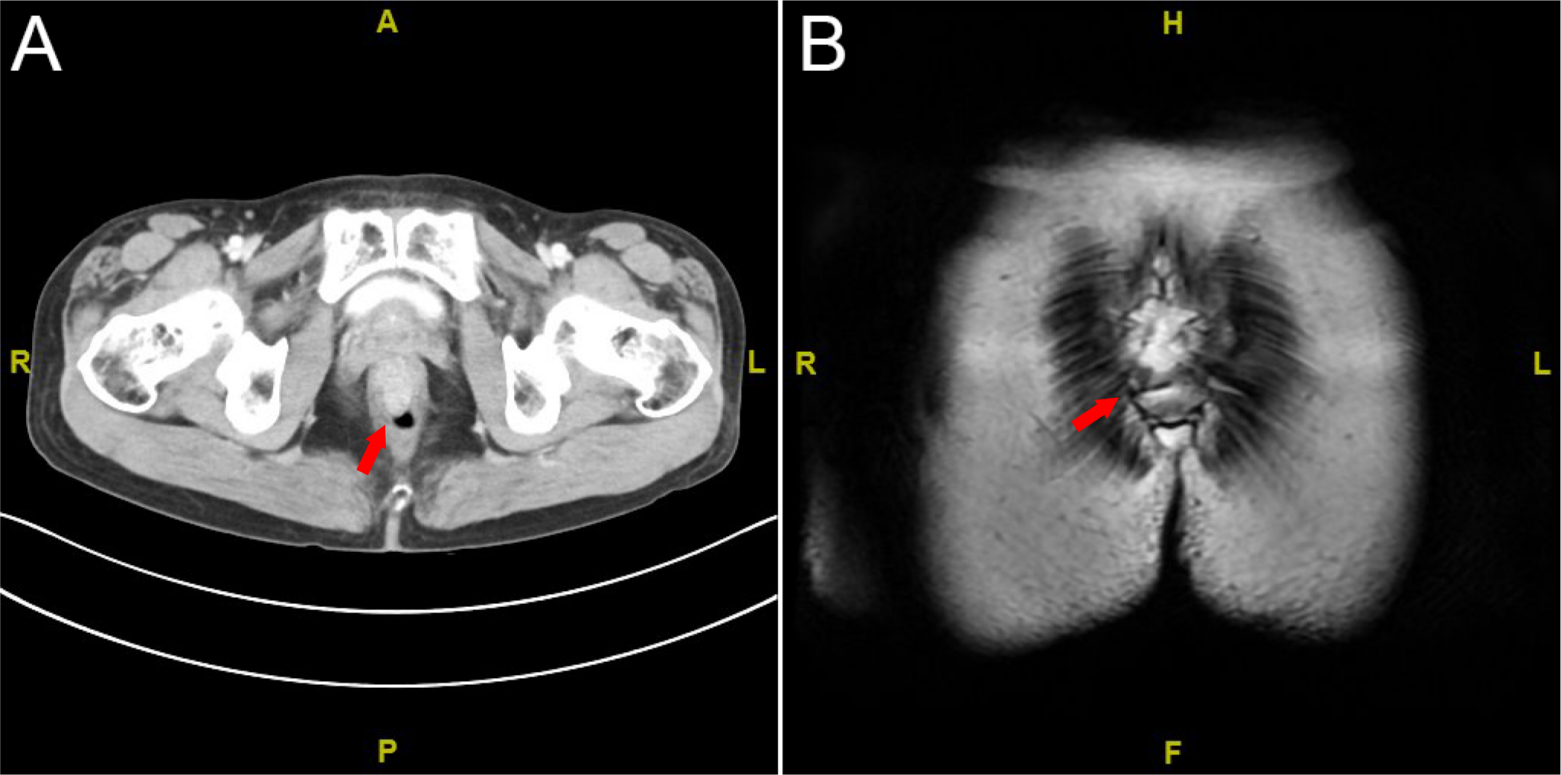
Imaging studies. **(A)** Contrast-enhanced abdominal CT scan; **(B)** pelvic MRI scan. The red arrow indicates the tumor.

Contrast-enhanced abdominal CT revealed thickening and abnormal enhancement of the distal rectal wall (**Figure 3A**). Pelvic MRI demonstrated an eccentric, heterogeneous soft tissue mass ~3.6 cm from the anal verge, exhibiting slightly prolonged T1 and T2 signals, restricted diffusion on DWI (high signal intensity), and penetration through the serosal surface. The lesion measured ~5.0 cm in length, causing luminal stenosis. Several small perirectal and presacral lymph nodes were noted. Dynamic contrast-enhanced imaging showed irregular linear enhancement of the presacral fascia. The heterogeneous lateral soft tissue mass at ~4.0 cm from the anal verge exhibited continuous and marked enhancement, with a slight reduction in enhancement intensity during the delayed phase. Adjacent perirectal and presacral lymph nodes displayed moderate, heterogeneous enhancement (**Figure 3B**).

### 2.5 Diagnosis and treatment

**Figure 4:**
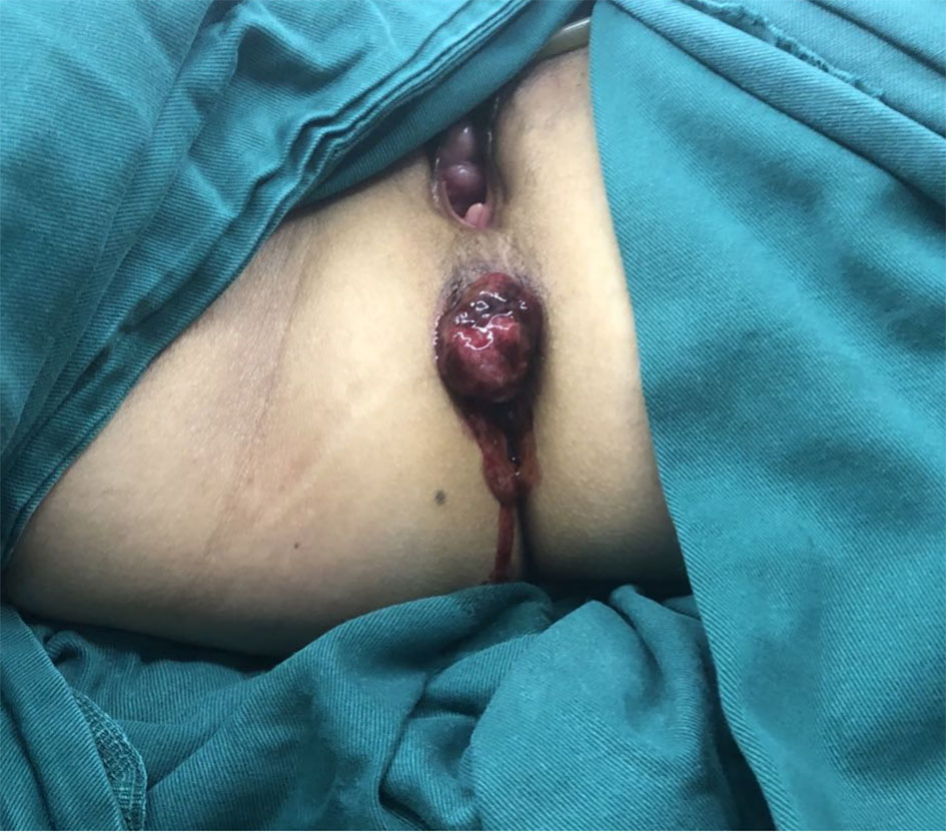
Intraoperative gross specimen of the tumor.

Based on clinical history and diagnostic investigations, the patient was diagnosed with ARMM. After excluding surgical contraindications, she underwent laparoscopic-assisted abdominoperineal resection (Miles procedure; **Figure 4**). Postoperative pathology report indicated malignant melanoma (anorectal region), measuring up to 3.0 cm in maximum diameter, with invasion into the muscular layer. No definite lymphovascular or perineural invasion was identified. The anal verge, bowel resection margins, and circumferential resection margin were free of tumor involvement. Surgical dissection revealed 13 perirectal lymph nodes, all negative for metastatic tumor. According to the AJCC Cancer Staging Manual, 8th Edition, the findings were consistent with pT2N0M0 staging. The patient was discharged in stable condition. Due to financial constraints and limited insurance coverage, the patient declined the recommended genetic testing and opted directly for adjuvant immunotherapy with toripalimab (240 mg every 2 weeks). No evidence of recurrence was observed on contrast-enhanced abdominal CT during the 3-month postoperative follow-up.

## 3 Discussion

Surgery is still the cornerstone of radical treatment for anorectal melanoma, a rare malignant tumor (8, 9). However, subsequent studies have shown comparable postoperative recurrence rates and overall survival (OS) with local and radical resection, suggesting that local resection may be a better option (10–13). Notwithstanding this, the CSCO Guidelines 2021 clearly state that for resectable anorectal melanoma, abdominoperineal resection (APR) remains the standard of care (14). In this case, given the absence of detected local or systemic metastasis and the patient’s relatively young age, where radical resection offered a potential for cure, the Miles procedure was selected. The patient responded equally positively to this suggestion.

With the growing application of adjuvant therapies, radiotherapy, chemotherapy, and immunotherapy have been increasingly integrated into ARMM management (15). Unfortunately, the long-term benefits of these modalities remain inconclusive (16–18). While adjuvant immunotherapy moderately prolongs OS in cutaneous melanoma, its efficacy in ARMM appears limited (18, 19). Current evidence only suggests that wide local excision combined with adjuvant therapy achieves survival outcomes comparable to radical resection while preserving sphincter function (15, 18). Nevertheless, we emphasize that adjuvant immunotherapy remains a rapidly evolving field. Studies indicate that PD-1 inhibitors (e.g., pucotenlimab) may still benefit certain patients with unresectable locally advanced or distantly metastatic mucosal melanoma (20).

Emerging evidence highlights the promise of neoadjuvant therapy combined with local or radical surgery. Compared to standard adjuvant regimens, neoadjuvant immunotherapy achieves 2-year recurrence-free survival rates of 70%–80% (21). A National Cancer Database retrospective study reported a 3-year OS of 61% with neoadjuvant therapy followed by local excision (11). In advanced-stage disease, PD-1 inhibitors combined with BRAF/MEK-targeted therapy significantly extend OS, particularly with the ipilimumab (anti-CTLA-4) and nivolumab (anti-PD-1) combination, which achieves a 5-year OS rate of 52% (22–24). Data from the International Neoadjuvant Melanoma Consortium (INMC) underscore the superiority of neoadjuvant immunotherapy over targeted therapy, yielding higher pathologic and radiologic response rates, as well as improved RFS and OS (25). These studies suggest that neoadjuvant therapy, especially neoadjuvant immunotherapy combined with surgery, may be a new direction to improve the prognosis of patients. Regrettably, the patient in this case declined preoperative neoadjuvant therapy due to financial constraints as well as fear of living with the tumor.

CAR-T therapy emerges as a novel mucosal melanoma treatment following molecular-targeted immunotherapies. Preclinical studies show CAR-T cells targeting Muc18/Tyrp1 effectively eradicate tumors and inhibit recurrence (26, 27). *In vitro*/*in vivo* studies confirm significant antitumor efficacy across diverse targets (28–31). Consequently, multiple clinical trials evaluate CAR-T efficacy (31). Despite challenges—off-target toxicity, antigen loss resistance, and relapse—innovative strategies (e.g., targeting TAAs, engineering chemokine receptors) show potential to overcome limitations (31, 32). However, currently, no specific CAR targets have been developed for ARMM, and related research remains in the early stages.

In conclusion, early detection, accurate diagnosis, and multidisciplinary individualized treatment are essential to improving the prognosis of patients with ARMM.

## 4 Conclusion

This case highlights hematochezia as the initial manifestation of ARMM and underscores the importance of early digital rectal examination and colonoscopy in elderly patients with gastrointestinal symptoms to mitigate diagnostic delays.

Despite the persistent challenges posed by ARMM’s rarity, diagnostic complexity, and limited therapeutic options—all of which hinder prognostic improvement—this case report offers valuable insight into the evolving management paradigm by meticulously documenting the complete diagnostic and therapeutic journey.

## Data Availability

The original contributions presented in the study are included in the article/supplementary material. Further inquiries can be directed to the corresponding authors.
